# Iceberg phenomenon in cutaneous leishmaniasis: A sporotrichoid clue

**DOI:** 10.1016/j.clinme.2025.100551

**Published:** 2026-01-02

**Authors:** Gokhan Okan, Mustafa Ozates

**Affiliations:** a Faculty of Medicine, Department of Skin and Venereal Diseases, Istanbul Aydin University, Istanbul, Türkiye; b HSM Radiology Advanced Imaging and Diagnositic Center, Istanbul, Türkiye

**Keywords:** *Leishmania major*, Meglumine antimoniate, Sporotrichoid leishmaniasis, MRI, Amphotericin B

## Abstract

We present a 29-year-old immunocompetent Uzbek woman with an ulcerated nodular lesion on her arm following an insect bite, caused by *Leishmania major* and confirmed by polymerase chain reaction (RT-PCR). The patient was initially treated with oral itraconazole and topical imiquimod. However, the disease progressed over 3 months and systemic liposomal amphotericin B was subsequently initiated.

Magnetic resonance imaging (MRI) revealed deep tissue involvement, marking the first such finding in sporotrichoid cutaneous leishmaniasis caused by *L major*. This case emphasises the diagnostic and treatment challenges of atypical cutaneous leishmaniasis and highlights the value of imaging techniques in assessing disease extent.

## Image report

A 29-year-old immunocompetent Uzbek woman presented to our clinic with a 2-month history of an ulcerated nodular lesion on her right arm following an insect bite during her visit to Uzbekistan. Dermatological examination revealed a 2 cm ulcerated nodule on the posteromedial aspect of her right distal arm. Laboratory tests, including HIV serology, were within normal limits. Polymerase chain reaction (RT-PCR) analysis of the tissue biopsy specimen confirmed *Leishmania major* infection. Histopathological analysis revealed an intense granulomatous dermal infiltrate; however, no leishmania bodies or characteristic asteroid bodies of sporotrichosis were observed. Fungal stains and fungal culture were negative.

**Fig. 1:**
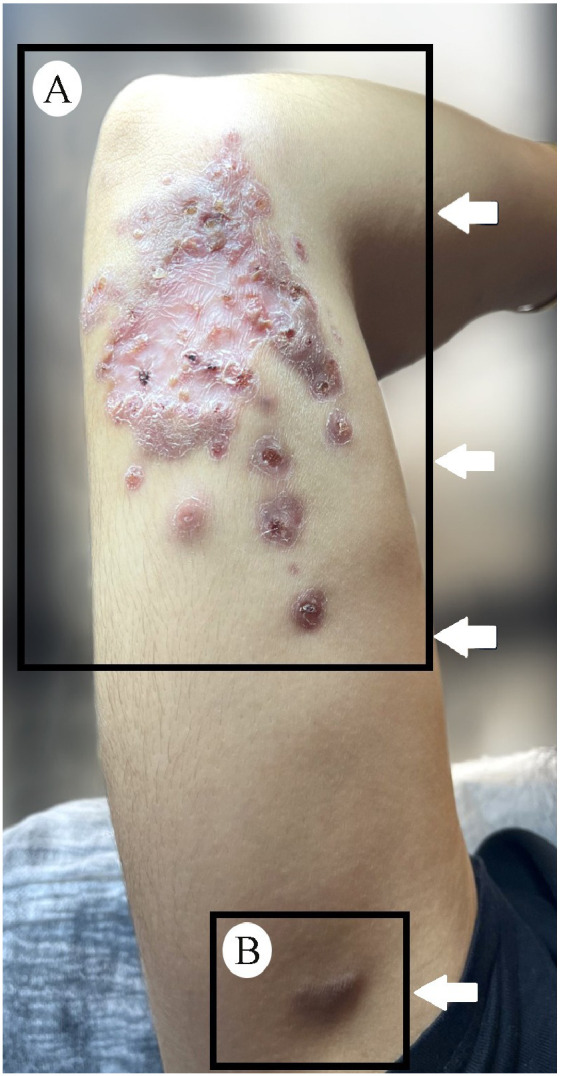
(A) Nodular plaque with central scarring and an active edge, accompanied by satellite lesions and three ulcerated nodules arranged linearly on the medial aspect of the arm with an additional lesion in the right axilla where (B) a subcutaneous nodule is also evident, suggestive of regional lymphadenopathy.

**Fig. 2:**
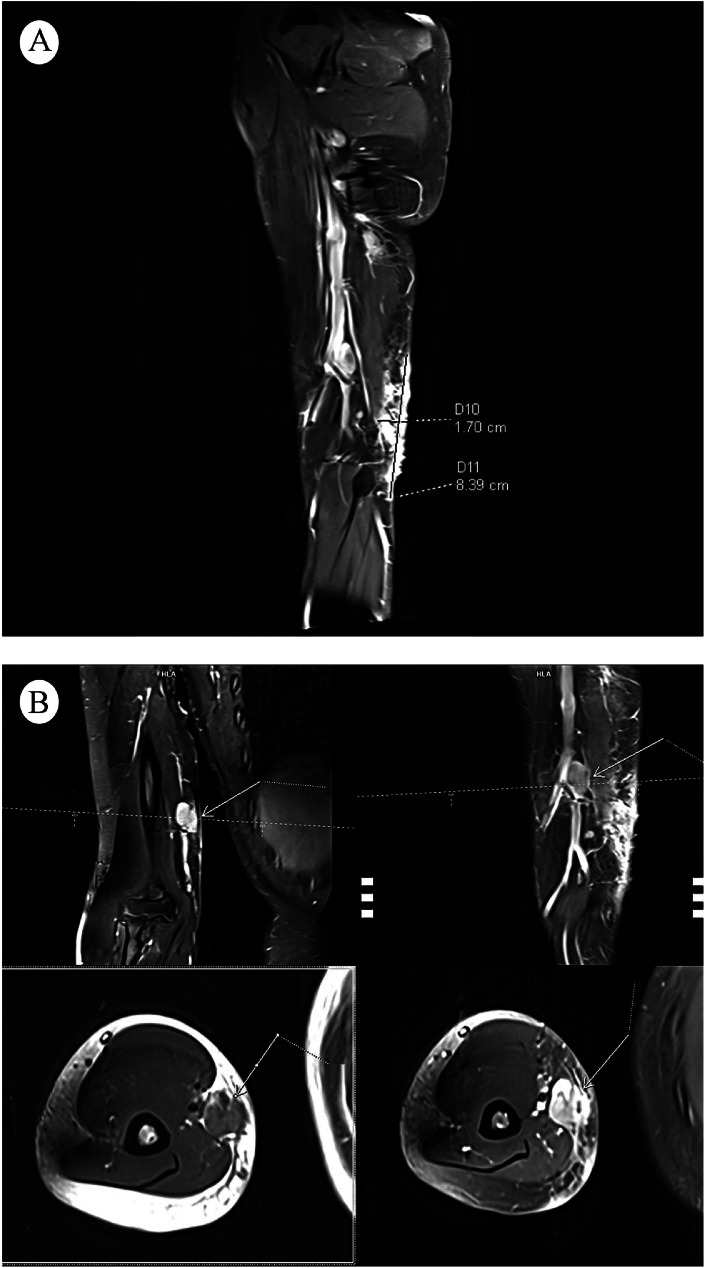
(A) Magnetic resonance imaging (MRI) of the upper extremity reveals a well-defined, oval-shaped lesion with hyperintense signal characteristics on T2-weighted sequences. The lesion is located in the soft tissue planes between muscle groups at the distal humerus level. (B) Mild surrounding oedema is noted on axial and coronal sections with regional and axillary lymphadenopathy.

Due to limited availability to meglumine antimoniate, oral itraconazole (200 mg/day) and topical imiquimod (three times weekly) were started. However, the disease progressed in 3 months; new subcutaneous nodules appeared in a linear pattern consistent with sporotrichoid spread (Fig. 1a). Magnetic resonance imaging (MRI) (Fig. 2) demonstrated oedema on the posteromedial aspect of her distal right arm, adjacent to the elbow joint, with regional and axillary lymphadenopathy (Fig. 1b). Therefore, systemic liposomal amphotericin B was administered intravenously at a dose of 3 mg/kg/day, once daily, for a total of five doses. Regression was observed at the 10th day, with no new lesions during 1-year follow-up.

Cutaneous leishmaniasis (CL) presents with a broad clinical spectrum ranging from classic ulcerative forms to atypical variants such as sporotrichoid, verrucous, annular, erysipeloid, discoid lupus-like, zosteriform, eczematous and carcinoma-like lesions.^1^ The clinical presentation depends on multiple factors, including the infecting *Leishmania* species, the host’s immune response, which is largely mediated through cellular immunity, as well as the site of infection, the number of parasites inoculated and nutritional status of the host. Sporotrichoid leishmaniasis is an atypical rare form of cutaneous leishmaniasis (CL) characterised by linear nodular lesions along lymphatic channels and regional lymphadenopathy, occurring either spontaneously or following local treatment-induced inflammation.^2^ In our case, the cutaneous leishmaniasis progressed despite treatment, transforming into the sporotrichoid form, for which systemic therapy with liposomal amphotericin B was initiated. If sporotrichoid leishmaniasis is left untreated, this form may progress to mucocutaneous involvement. MRI assessment of the lesion depth in sporotrichoid leishmaniasis provided crucial information for evaluating the risk of progression to mucocutaneous involvement.

Itraconazole is a limited alternative for the treatment of cutaneous leishmaniasis caused by certain *Leishmania* species due to its low cost, ease of administration and favourable safety profile; however, its activity against *L major* is variable and often similar to placebo, which is consistent with the treatment failure observed in our case.^3^ Local inflammation may have facilitated the lymphatic spread of the parasite. Liposomal amphotericin B remains the most effective systemic therapy for *L major* and *Leishmania tropica*, though its use is constrained by high cost and potential nephrotoxicity.^4^

Disseminated and complex forms of CL, particularly those with lymphatic spread, require close monitoring due to risk of treatment failure or progression to mucocutaneous disease, which may occur despite the absence of drug resistance.

Although these complications are more frequently observed in immunosuppressed individuals, sporadic cases have also been reported in immunocompetent patients, including infections caused by *L major*.^5^ Treatment outcomes are influenced by the infecting *Leishmania* species, host immune status and pharmacologic variables.^6^ Early recognition of these forms is crucial, as local therapies may be insufficient, and systemic treatment may be required. Following systemic treatment with liposomal amphotericin B, clinical regression was observed by the 10th day in our patient and no new lesions developed during the 1-year follow-up.

This case highlights the diagnostic and therapeutic challenges of sporotrichoid leishmaniasis caused by *L major*. To our knowledge, this is the first reported case of sporotrichoid leishmaniasis with MRI-documented deep tissue involvement.

## Consent for publication

Written informed consent for publication was obtained from the patient.

## Funding

This research did not receive any specific grant from funding agencies in the public, commercial or not-for-profit sectors.

## CRediT authorship contribution statement

**Gokhan Okan:** Writing – review & editing, Methodology, Data curation, Conceptualization. **Mustafa Ozates:** Writing – original draft, Visualization, Resources.

## Declaration of competing interest

The authors declare that they have no known competing financial interests or personal relationships that could have appeared to influence the work reported in this paper.
